# Genomic selection of reference genes for real-time PCR in human myocardium

**DOI:** 10.1186/1755-8794-1-64

**Published:** 2008-12-29

**Authors:** Anna P Pilbrow, Leigh J Ellmers, Michael A Black, Christine S Moravec, Wendy E Sweet, Richard W Troughton, A Mark Richards, Chris M Frampton, Vicky A Cameron

**Affiliations:** 1 Christchurch Cardioendocrine Research Group, Department of Medicine, University of Otago-Christchurch, PO Box 4345, Christchurch 8014, New Zealand; 2 Department of Biochemistry, University of Otago, PO Box 56, Dunedin 9054, New Zealand; 3 Kaufman Center for Heart Failure, Department of Cardiovascular Medicine, Cleveland Clinic, 9500 Euclid Avenue, Cleveland, Ohio 44195, USA

## Abstract

**Background:**

Reliability of real-time PCR (RT-qPCR) data is dependent on the use of appropriate reference gene(s) for normalization. To date, no validated reference genes have been reported for normalizing gene expression in human myocardium. This study aimed to identify validated reference genes for use in gene expression studies of failed and non-failed human myocardium.

**Methods:**

Bioinformatic analysis of published human heart gene expression arrays (195 failed hearts, 16 donor hearts) was used to identify 10 stable and abundant genes for further testing. The expression stability of these genes was investigated in 28 failed and 28 non-failed human myocardium samples by RT-qPCR using geNorm software.

**Results:**

Signal recognition particle 14 kDa (SRP14), tumor protein, translationally-controlled 1 (TPT1) and eukaryotic elongation factor 1A1 (EEF1A1) were ranked the most stable genes. The commonly used reference gene, glyceraldehyde-3-phosphate dehydrogenase (GAPDH) was ranked the least stable of the genes tested. The normalization strategy was tested by comparing RT-qPCR data of both normalized and raw expression levels of brain natriuretic peptide precursor (NPPB), a gene known to be up-regulated in heart failure. Non-normalized levels of NPPB exhibited a marginally significant difference between failed and non-failed samples (p = 0.058). In contrast, normalized NPPB expression levels were significantly higher in heart-failed patients compared with controls (p = 0.023).

**Conclusion:**

This study used publicly available gene array data to identify a strategy for normalization involving two reference genes in combination that may have broad application for accurate and reliable normalization of RT-qPCR data in failed and non-failed human myocardium.

## Background

Analysis of gene expression levels has become increasingly important in biological research, providing insight into the complex regulatory networks that underlie health and disease [1]. Quantitative real-time PCR (RT-qPCR) is one of the most sensitive, specific, and widely-used methods for measuring the expression levels of selected genes [2,3]. Accuracy of RT-qPCR data is reliant on appropriate normalization of individual samples, and sub-optimal normalization is a common pitfall of RT-qPCR analyses [1,2,4]. There are many sources of variation in gene expression measurements, including differences in cell number, transcriptional efficiency, mRNA integrity, and differences in efficiency of RNA extraction and variability of cDNA synthesis between samples. While a number of strategies have been applied to account for inter-sample variation, the use of two or more reference genes is currently the favored approach for accurate and reliable normalization of the mRNA fraction [2,5].

Among the most commonly used reference genes in RT-qPCR are glyceraldehyde-3-phosphate dehydrogenase (GAPDH) and β-actin [6]. These genes were introduced for normalization when mRNA expression was assessed using semi-quantitative methods, such as Northern blotting and RNase protection, because of their relatively high abundance in numerous cell-types [1,4]. However, the increased sensitivity of RT-qPCR demands a significantly greater stringency for reference gene selection [2,5]. Previous studies have demonstrated that the expression levels of classical reference genes can vary markedly across cells, tissues and experimental conditions, including heart cells and tissues [7,8]. More stable alternatives exist, although these tend to be specific to the tissue under investigation [1,6,9,10]. To date, no validated universal or human heart-specific reference genes have been identified.

To select optimal reference genes for RT-qPCR analysis of human myocardium, we screened human heart gene expression data from a published microarray study and investigated gene abundance and expression stability. We validated the top-ranked candidate reference genes in an independent set of human myocardium samples obtained from heart transplant patients and heart-healthy donors, and identified the minimum set required for accurate and reliable normalization of RT-qPCR data in failed and non-failed human myocardium.

## Methods

### Candidate gene selection

To select candidate reference genes for subsequent testing by RT-qPCR, publicly available microarray data from explanted human myocardium was screened to identify transcripts with stable and abundant expression. Abundant transcripts were selected to ensure robust measurement by RT-qPCR in all samples. Affymetrix gene expression profiles generated from left ventricle myocardium from 195 heart transplant recipients with advanced ischemic or idiopathic cardiomyopathy and 16 unmatched heart donors [11] were obtained from the NCBI GEO database (GEO accession GSE5406, ). These data were derived from Affymetrix HG-U133A microarrays, each consisting of 22,283 probe sets representing approximately 13,000 genes. MAS 5.0 [12] normalized data were used to calculate the mean and standard deviation of the expression intensity for each probe set across all samples, using the R software package [13]. The 5% of probe sets with the smallest standard deviations (i.e., the least variable) were then identified, and of those, any probe set with a mean expression intensity of greater than 10 on the log2 scale was selected for assessment as a candidate reference for RT-qPCR analysis. For comparison, the expression stability and abundance of 50 genes previously used or proposed by prior studies as reference genes [6,10,14] were also screened for abundance and stability across all probes for identification of candidate reference genes.

### Patient samples

Tissue from the left ventricular free wall of the myocardium was obtained from explanted failed hearts from heart transplant recipients at the Cleveland Clinic Foundation (n = 28). Non-failed heart tissue was obtained from unmatched organ donors through Lifebanc of Northeast Ohio (n = 28). Tissue handling was as previously described [15,16]. The investigation was approved by the Cleveland Clinic Internal Review Board (ethics approval IRB 2378) and all patients provided informed consent. The study adhered to the principles outlined in the Declaration of Helsinki and Title 45, US Code of Federal Regulation, Part 46, Protection of Human Subjects, revised November 13, 2001, effective December 13, 2001. All procedures followed were in accordance with institutional guidelines.

### Sample preparation

Frozen tissue blocks (previously stored at -80°C, mean weight ± standard deviation = 181 ± 81 mg) were placed in pre-chilled tubes containing TRIzol^® ^(Invitrogen, Carlsbad, CA) and immediately subjected to automated grinding in a Mixer Mill MM301 (Retsch, Haan, Germany). Total RNA was isolated with chloroform and purified using RNeasy Midi columns in a total volume of 300 μL (Qiagen, Valencia, CA) according to the manufacturer's instructions. RNA yield and purity was determined using a Nanodrop spectrophotometer (Nanodrop Technologies, Montanin, DE). The mean RNA concentration and 260:280 ratio (± standard deviation) was 749 ± 271 ng/μL and 2.05 ± 0.03, respectively. The integrity of the RNA was assessed with gel electrophoresis. All samples were digested with DNase I (Invitrogen, Carlsbad, CA). First strand cDNA synthesis was performed from 2 μg of total RNA with oligodT primers and Superscript III, followed by RNase H digestion (Invitrogen, Carlsbad, CA) as previously described [17].

### Real-time PCR

Primers for all 10 candidate reference genes were designed using Primer3 software [18] within regions spanned by the Affymetrix probe set target sequences (Table 1). At least one primer in every primer pair was designed across an exon/exon boundary to prevent amplification of any dsDNA transcripts not removed by digestion with DNase I. Affymetrix probe sequences were obtained from the Affymetrix NETAFFX Analysis Centre . Alignments of Affymetrix probe set target sequences with mRNA and genomic DNA sequences for each gene were performed with Genious version 2.0.01 software. RT-qPCR reactions were performed in duplicate in a Rotor-Gene 3000 (Corbett Research, Sydney, Australia) using SYBR Green I detection of dsDNA synthesis. Reactions (20 μL) contained 1 μL template cDNA, 1× PCR buffer, 1.5 mM MgCl2, 0.2 mM dNTPs (Fermentas, Glen Burnie, MD), 0.5 mM forward and reverse primers, 5× SYBR Green I (Roche Diagnostics, Mannheim, Germany) and 1 U Taq-Ti DNA polymerase (Fisher Biotec, West Perth, Australia). The cycling conditions comprised 2 mins polymerase activation at 94°C followed by 30 cycles of 94°C for 10s, 56°C for 20s, and 68°C for 10s. On completion, amplimers were exposed to a temperature gradient from 79–95°C (melt curve) to confirm that a single product had been amplified. For each sample the Ct value (the fluorescent point at which the reactions are compared) was fitted to a standard curve consisting of five serial dilution points (in triplicate) of purified DNA template (amplicon derived, copy number ranging from ~109 to ~105 copies) and a no-template control. The reaction efficiency for each primer set is detailed in Table 1. The mean standard deviation of Ct values for duplicated samples was 0.10. Quantification of each sample was performed using Rotor-Gene software version 6.1.

**Table 1:** Real-time PCR primers

**Primer**		**Sequence**	**Tm**	**Length (bp)**	**Efficiency (%)**
GAPDH	Forward	GCTCATTTCCTGGTATGACAACG	63	213	92.8
	Reverse	AGGGGTCTACATGGCAACTG	60		
RPL22	Forward	CCATGGCTCCTGTGAAAAAG	61	219	91.6
	Reverse	TCACGGTGATCTTGCTCTTG	60		
TPT1	Forward	AAATGTTAACAAATGTGGCAATTAT	58	164	95.9
	Reverse	AACAATGCCTCCACTCCAAA	61		
RPS4X	Forward	GATCCCCTCATCAAGGTGAA	60	243	78.7
	Reverse	GCCCTTGCCAATAACAAAAA	60		
RPL13A	Forward	CGCCCTACGACAAGAAAAAG	60	206	96.9
	Reverse	CCGTAGCCTCATGAGCTGTT	60		
RPL23A	Forward	GCTCCCAGGAGAAACAAGC	60	201	92.2
	Reverse	ATCAGGCCGAATCAGGGTGTT	65		
EEF1A1	Forward	CTTTGGGTCGCTTTGCTGTT	63	183	95.0
	Reverse	CCGTTCTTCCACCACTGATT	60		
RPL41	Forward	ATGAGAGCCAAGTGGAGGAA	60	219	94.0
	Reverse	TCAGAGGGCGATGAAGTTCT	60		
RNPS1	Forward	ACCCATGGTAGTTGCTGCTC	60	104	95.3
	Reverse	AGCTGGCTCTCCACTCACTC	60		
SRP14	Forward	CAGATGGCTTATTCAAACCTCCT	61	181	99.9
	Reverse	ATGCCCTTTACTGTGCTGCT	60		

NPPB expression levels were determined using a Taqman gene expression assay with inventoried probes (assay id # Hs00173590_m1, Applied Biosystems, Foster City, CA). Reactions (20 μL) were performed in triplicate on a 7500 Fast real-time PCR system (Applied Biosystems) in standard mode according to manufacturer's instructions. Samples were quantified using a standard curve consisting of five serial dilution points (in triplicate) of purified DNA template (amplicon derived, copy number ranging from 1.13 × 108 – 1.13 × 104 copies) and a no-template control. Reaction efficiency was 101.2%. The mean standard deviation of Ct values for duplicate samples was 0.16. Quantification was performed with 7500 Software version 2.0 (Applied Biosystems).

### Evaluation of candidate reference genes using GeNorm

The Visual Basic Application geNorm version 3.4 [5,14] was used to evaluate the expression stability of each gene, determine the ideal number of genes required for normalization, and calculate individual normalization factors based on the expression levels of the best reference genes. In geNorm analysis, the calculated gene stability measure (M) relies on the principle that the ratio of two ideal reference genes will be identical in all samples. M is calculated as the average pairwise variation between a particular gene and all other genes in the analysis. Decreasing expression stability occurs when one or more of the genes is not constantly expressed. At each step in the analysis the least stable gene (highest M-value) is excluded and M is recalculated. This process continues in a step-wise manner until the two most stable genes remain. To determine the optimum number of genes for normalization, the two most stable genes are used as a starting point. Genes are added sequentially (from most stable to least stable) until the pairwise variation between two sequential normalization factors drops below a set threshold, indicating that the prior gene set is sufficient for accurate normalization [5]. A large variation indicates that the added gene has a significant effect and should preferably be included, provided it is stably expressed. However, genes that are less stably expressed also give high pairwise variation because they have a different pattern of expression compared with the more stably expressed genes. Therefore, to achieve accurate normalization, less stable genes should not be included in calculation of the normalization factor, despite their high pairwise variation values. Based on their findings Vandesompele et. al. recommended setting the pairwise variation threshold at 0.15 [5].

Using the geNorm algorithm, normalization factors were calculated by transforming Ct values into quantities (using standard curves or the comparative Ct method), setting the highest relative quantity for each gene to 1, and scaling the expression value for all other samples to a proportion relative to this highest value. Normalization factors using multiple genes were determined by calculating the geometric mean of the scaled reference genes.

### Statistical analyses

Univariate analyses were performed using analysis of variance to compare gene expression levels in heart-failed and non heart-failed groups. Gene expression data displayed consistently skewed distributions and hence these data were log-transformed and geometric means with 95% confidence intervals have been reported. Correlations between expression levels of ribosomal genes and between normalization factors generated with two, three, four or five genes were calculated using Pearson correlation coefficients. All statistical analyses were performed with SPSS version 16 (SPSS Inc., Chicago, IL). A p-value < 0.05 was taken to indicate statistical significance.

## Results

**Figure 1:**
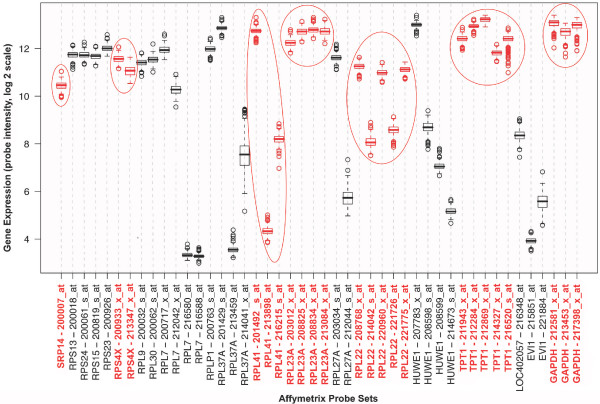
**Selection of candidate reference genes**. Expression levels of probe sets for genes with one or more probe sets among the top 20 most stable and abundantly expressed across all samples, identified from screening publicly available Affymetrix gene expression profiles of left ventricle myocardium from 195 heart transplant recipients and 16 unmatched heart donors [11] (GEO accession GSE5406, ). GAPDH, RPL22, RPS4X, TPT1, RPL23A, RPL41 and SRP14 genes were selected as candidate reference genes (shown in red), based on their high abundance and consistent expression across the majority of probe sets. Affymetrix probe set nomenclature is preceded by the gene symbol. Boxes indicate median and interquartile range, whisker length is 150% of the interquartile range. Observations beyond the whiskers are denoted by open circles.

Twenty genes were identified as being represented by at least one probe set exhibiting low variation and high abundance among all samples (Figure 1). From this analysis 7 genes were selected as candidate reference genes for further testing with RT-qPCR based on whether other probe sets for that gene also had small variance and high abundance across samples. The genes selected were GAPDH, RPL22, RPS4X, TPT1, RPL23A, RPL41 and SRP14 (see Table 2 for full gene name, chromosomal location, function and expression levels in heart-failed and control tissue). In addition, analysis of 50 genes previously used or proposed by prior studies as reference genes [6,10,14] identified an additional three genes with small variance across probes: RPL13A, EEF1A1 and RNPS1 (Table 2, see Additional file 1 for Affymetrix probe intensities for all 50 genes). Thus a total of 10 genes were selected as candidates for testing, of which 4 encoded ribosomal proteins (RPL22, RPL23A, RPL41 and RPL13A) and are involved in protein biosynthesis. Because the expression levels of the ribosomal genes were tightly correlated (Table 3) and co-regulation of genes may confound geNorm analyses, only one of the ribosomal proteins could be included in the analysis. A preliminary analysis identified RPL13A as the most stable ribosomal gene for inclusion in the geNorm analysis (data not shown). The remaining ribosomal genes were excluded. There was no relationship between the functional class of any of the other genes (Table 2).

**Table 2:** Candidate cardiac reference genes ranked in order of abundance

**UniGene accession**	**Gene symbol**	**Gene name**	**Gene ontology biological process**	**Location**	HF patients* (copy #/μg total RNA)	Controls* (copy #/μg total RNA)	**p-value (HF vs controls)**
Hs.479728	GAPDH	glyceraldehyde-3-phosphate dehydrogenase	glucose metabolism, glycolysis, translational initiation, cell communication,	12p13	1.13 × 10^9^ (0.91 – 1.40 × 10^9^)	1.65 × 10^9^ (1.33 – 2.05 × 10^9^)	0.015
Hs.515329	RPL22	ribosomal protein L22	protein biosynthesis	1p36	1.48 × 10^7^ (1.13 – 1.95 × 10^7^)	2.80 × 10^7^ (2.13 – 3.68 × 10^7^)	0.002
Hs.374596	TPT1	tumor protein, translationally-controlled 1	calcium and microtubule-binding	13q12	1.00 × 10^7^ (0.60 – 1.69 × 10^7^)	1.93 × 10^7^ (1.15–3.26 × 10^7^)	0.080
Hs.446628	RPS4X	ribosomal protein S4, X-linked	regulation of cell cycle, protein biosynthesis, development, cell proliferation	Xq13	0.89 × 10^7^ (0.62 – 1.27 × 10^7^)	1.99 × 10^7^ (1.39 – 2.86 × 10^7^)	0.003
Hs.523185	RPL13A	ribosomal protein L13a	protein biosynthesis	19q13	2.33 × 10^6^ (1.39–3.90 × 10^6^)	6.24 × 10^6^ (3.73 – 10.45 × 10^6^)	0.009
Hs.419463	RPL23A	ribosomal protein L23a	protein biosynthesis	17q11	2.44 × 10^6^ (1.78 – 3.34 × 10^6^)	5.51 × 10^6^ (4.02 – 7.56 × 10^6^)	0.001
Hs.490287	EEF1A1	Eukaryotic elongation factor 1A1	translational elongation	6q14	2.44 × 10^6^ (1.42 – 4.17 × 10^6^)	4.47 × 10^6^ 2.61–7.65 × 10^6^	0.116
Hs.112553	RPL41	ribosomal protein L41	protein biosynthesis	12q13	1.78 × 10^6^ (1.08 – 2.93 × 10^6^)	4.70 × 10^6^ (2.86 – 7.73 × 10^6^)	0.008
Hs.355643	RNPS1	RNA binding protein S1	transcription, RNA splicing	16p13	6.19 × 10^5^ (4.04 – 9.48 × 10^5^)	11.00 × 10^5^ (7.19 – 16.85 × 10^5^)	0.061
Hs.533732	SRP14	signal recognition particle 14 kDa	protein targeting	15q22	4.03 × 10^5^ (2.30 – 7.08 × 10^5^)	10.76 × 10^5^ (6.14 – 18.90 × 10^5^)	0.017

* Transcript copy number per μg total RNA, geometric mean (95% confidence interval). HF = heart failure

**Table 3:** Pearson correlations of expression levels of ribosomal genes

	**RPL13A**	**RPL22**	**RPL23A**	**RPL41**
**RPL13A**	1.00	0.851 p < 0.001	0.866 p < 0.001	0.952 p < 0.001
**RPL22**		1.00	0.886 p < 0.001	0.831 p < 0.001
**RPL23A**			1.00	0.903 p < 0.001
**RPL41**				1.00

**Figure 2:**
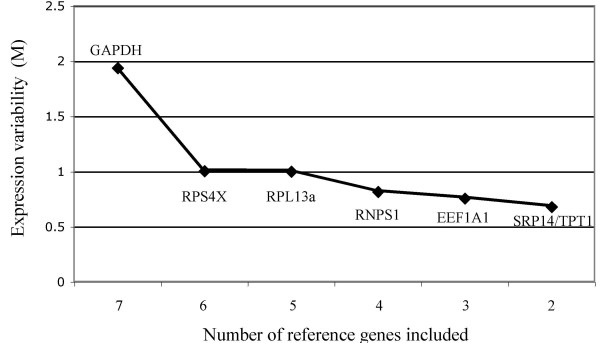
**Average expression variability of reference genes (M), during stepwise exclusion of the least stable gene**. At each step the most variable gene (highest M-value) is excluded and M is recalculated. This process continues in a step-wise manner until the two most stable genes remain. The figure indicates that GAPDH is least stably expressed relative to the other genes, and that SRP14 and TPT1 have the most stable expression.

**Figure 3:**
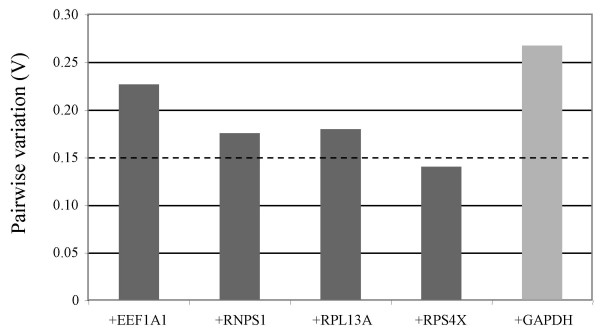
**Determination of the ideal number of reference genes for normalization**. Using the two most stable genes as a starting point (SRP14 and TPT1), genes are added sequentially (from most stable to least stable) until the pairwise variation between two sequential normalization factors drops below the recommended threshold of 0.15, indicating that the prior gene set is sufficient for accurate normalization. Variation greater than 0.15 indicates that the added gene has a significant effect and should preferably be included, provided it is stably expressed. This figure shows that the sixth most stable gene (RPS4X) is not required for reliable normalization of RT-PCR data.

Genes were ranked from least stable to most stable by geNorm analysis: GAPDH, RPS4X, RPL13A, RNPS1, EEF1A1, TPT1/SRP14 (Figure 2). All genes had high expression stability (M-values <1), below the default limit of M = 1.5, except for GAPDH. GAPDH was considerably more variably expressed (M-value = 1.94). GeNorm analysis indicated that the top five most stable genes would be needed for reliable normalization of RT-qPCR data (geNorm recommended threshold = 0.15, Figure 3).

Univariate analysis showed that the expression levels of all candidate reference genes except for TPT1 (p = 0.080), EEF1A1 (p = 0.116) and RNPS1 (p = 0.061) were significantly lower in the myocardium of end-stage heart failure patients compared with non heart-failed donors (Table 2). Thus, these genes have utility as a reference within heart-failed myocardium samples or non-failed donor myocardium samples separately, but not for comparing gene expression between these groups. Notably expression levels of the commonly used reference gene, GAPDH, differed significantly between failed and non-failed myocardium (p = 0.015, Table 2).

**Figure 4:**
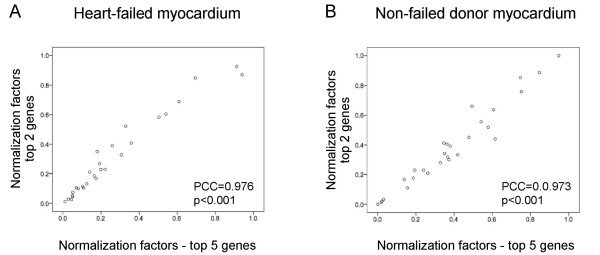
**Correlation of individual normalization factors generated using the top five most stable genes and the top two most stable genes**. Tight correlation between normalization factors indicates that as few as two genes may be sufficient for reliable normalization of RT-qPCR data for (A) heart-failed myocardium and (B) non-failed donor myocardium. PCC = Pearson correlation coefficient.

Within heart-failed and non-failed myocardium samples separately, we explored whether fewer than five reference genes could be used for normalization. A series of normalization factors were generated for each sample using geometric means of the top two, three or four most stable genes and correlated with the original set of normalization factors generated using all five top-ranked genes. Very tight correlation was observed between normalization factors for all gene sets (Pearson correlation coefficients, heart-failed samples: 0.976–0.995, p < 0.001; non-failed controls: 0.973–0.992, p < 0.001). Figure 4 illustrates the tight correlation between normalization factors generated with the top two (SRP14 and TPT1) and the top five most stable genes, indicating that as few as two genes may be sufficient for reliable normalization within failed and non-failed myocardium. Normalization factors generated using SRP14 and TPT1 were not correlated with GAPDH levels, in failed myocardium (Pearson correlation coefficient = -0.153, p = 0.436) or non-failed myocardium (Pearson correlation coefficient = -0.181, p = 0.356), suggesting that normalization of gene expression data with GAPDH in these samples would be inappropriate.

The expression levels of TPT1, EEF1A1 and RNPS1, did not differ significantly between failed and non-failed human myocardium samples (Table 2). These genes ranked among the top four most stable genes of those tested (Figure 2). Normalization of gene expression in failed and non-failed myocardium using these genes was tested by comparing normalized and raw expression levels of NPPB, a gene known to be up-regulated in heart-failed heart tissue. In the raw data NPPB levels were higher in heart-failed patients compared with controls, but the difference did not reach statistical significance (p = 0.058, Table 4). In normalized data, NPPB levels were significantly higher in heart-failed patients compared with controls (p = 0.023, Table 4). Removing the least stable gene, RNPS1, from the pool of reference genes only marginally affected the results (p = 0.024, Table 4). Mean NPPB levels were almost identical to data normalized with RNPS1 included, indicating that as few as two genes (TPT1 and EEF1A1) may be sufficient for reliable normalization. Normalization factors generated using TPT1, EEF1A1 and RNPS1 were not correlated with GAPDH levels (Pearson correlation coefficient = -0.085, p = 0.535). Normalizing NPPB to GAPDH alone gave a larger difference between failed and non-failed myocardium (4.2-fold, p = 0.012, Table 4) compared with data normalized with TPT1, EEF1A1 and RNPS1 combined (3.4-fold, p = 0.023, Table 4).

**Table 4:** Normalization of NPPB using fewer than three reference genes

**Genes used for normalization**	**Correlation with normalization factors using TPT1, EEF1A1 & RNPS1**		**Normalized NPPB levels (geometric mean and 95% CI, copy #/μg total RNA)**		
	*PCC*	*p-value for correlation*	*HF patients*	*Controls*	*p-value (HF vs controls)*

TPT1, EEF1A1 & RNPS1	1.000	-	11.3 × 10^6^ (5.3 – 24.0 × 10^6^)	3.3 × 10^6^ (1.5 – 6.9 × 10^6^)	0.023
TPT1 & EEF1A1	0.989	<0.001	11.2 × 10^6^ (5.3 – 23.9 × 10^6^)	3.3 × 10^6^ (1.5 – 7.0 × 10^6^)	0.024
GAPDH	-0.085	0.535	77.5 × 10^6^ (35.6 – 168.9 × 10^6^)	18.5 × 10^6^ (8.5 – 40.4 × 10^6^)	0.012
Raw data	-	-	14.1 × 10^6^ (6.6 – 30.4 × 10^6^)	4.9 × 10^6^ (2.3 – 10.6 × 10^6^)	0.058

PCC = Pearson correlation coefficient, HF = heart failure

## Discussion

Validation of tissue-specific reference genes is a fundamental first-step in RT-qPCR analysis. We have identified two genes, SRP14 and TPT1, that in combination can be used for accurate and reliable normalization of RT-qPCR gene expression data within failed human myocardium and non-failed human myocardium separately, and two genes, TPT1 and EEF1A1 that are more stably expressed than GAPDH and may be used for normalization of RT-qPCR data when comparing gene expression levels between failed and non-failed human myocardium samples.

The physiological functions of SRP14, TPT1 and EEF1A1 relate to important independent cellular processes, emphasizing their utility as reference genes. SRP14 is a component of the signal recognition particle, a universally conserved ribonucleoprotein complex that mediates targeting of membrane and secretory proteins to the endoplasmic reticulum [19]. TPT1 encodes the abundant and highly conserved translationally controlled tumor protein (TCTP), which is reported to play a role in cell growth and cell cycle progression [20]. EEF1A1 is a component of the alpha subunit of the elongation factor-1 complex, which facilitates the enzymatic delivery of aminoacyl tRNAs to the ribosome [21]. Notably, TPT1 is expressed in a tissue- and development-specific manner, suggesting that its utility as a reference gene in human myocardium may be limited to analyses in adult hearts [22].

To date, reference genes have been identified for a wide range of species, tissue types, experimental conditions and disease states. The typical methodological approach used in these studies has been to test a selection of commonly used reference genes such as GAPDH, β-actin, α-tubulin, hypoxanthine phosphoribosyl-transferase 1 and 18S RNA to identify the most stable combination specific to the sample and experimental conditions under investigation [23-29]. A small number of studies have reported that none of the traditional reference genes tested were appropriate for use in the particular setting investigated [30-32]. More recently, a genome-wide approach has been employed, where candidate reference genes selected from publicly available microarray data has lead to identification of novel transcripts that are more stably expressed than commonly used reference genes. These studies can be broadly classified into two groups: those that screen expression data from a diverse range of tissue types and species to identify transcripts that are stably and ubiquitously expressed and may therefore have board utility as reference genes [6,10,33-35], and those that screen array data from a specific tissue or disease setting to identify a set of candidate genes for validation in an independent sample set, as in the present study. To date, studies in the latter group have validated panels of novel reference genes in human normal and cancerous lung tissues [36,37], human normal and tumor gastrointestinal tissues [38], human embryonic stem cells [39], porcine mammary tissue [40], canine osteoarthritic joint tissue [41], barley [42] and lymphoblastoid cell lines and fibroblasts [43]. Notably, Peltier et. al. have identified a set of microRNA references for use in RT-qPCR analysis of microRNAs in normal and cancerous human solid tumors [44]. However, no validated universal reference genes have been identified.

In the present study, screening of Affymetrix cardiac expression data enabled us to identify four novel genes and six genes commonly used, or recommended for use, as references in human tissue, as potentially suitable for use in heart tissue RT-qPCR analyses. Interestingly, there were striking similarities between the genes we identified as stably expressed in cardiac microarrays and those identified in a recent meta-analysis of 13,629 human gene arrays of a diverse range of cell types and experimental conditions [6]. Both studies identified SRP14, RPL13A and RPL22 as being highly stably expressed, and found an over-representation of ribosomal genes among the top ranked transcripts. However, we also identified novel candidate genes specific to heart tissue, including TPT1, one of the most stably expressed genes in our analysis. Our finding that all genes selected were more stably expressed than GAPDH is consistent with an analogous study in gastrointestinal normal and tumor tissues [38], where GAPDH was the less stably expressed genes in tissue from stomach, small intestine, liver, and lymph nodes compared with expression of 8 candidate reference genes selected from analysis of publicly available Affymetrix array data.

GeNorm [5] is one of several statistical algorithms that evaluate the relative expression stability of genes on the basis of non-normalized expression levels. Other programs for assessing gene stability include Best keeper [45], NormFinder [46], Global Pattern Recognition [47] and equivalence tests [48]. These programs have provided a rational basis for selection of reference genes for normalization of RT-qPCR data. While the relative gene stability rankings may vary subtly depending on which algorithm is applied [41,49,50], use of multiple reference genes provides a considerably more robust result compared with relying on a single RNA transcript [5]. This reflects the heterogeneity inherent in human samples related to age, gender, ethnicity, and gene-environment interactions. In the present study all donors were on life-support as a result of head trauma or cerebral vascular accident and thus the gene expression profile of the donor heart samples may have been affected by the traumatic events and acute drug treatments that preceded the donation of tissue. Similarly, gene expression in heart-failed tissue will vary depending on the etiology and duration of heart failure and chronic and acute drug treatments.

GeNorm analysis enables the ideal number of reference genes required for reliable normalization to be determined. Our analysis indicated that the top five genes would be required for accurate normalization of RT-qPCR data within failed and non-failed human myocardium, however, the number of genes used needs to be a balance between accuracy and practical considerations. Realistically, if two relatively stable genes have been identified, it is unnecessary to add more genes if the normalization factor does not change markedly when they are included. We were able to demonstrate that as few as two genes may be sufficient for normalization in human myocardium (SRP14 and TPT1 for analysis within failed or non-failed tissue and TPT1 and EEF1A1 for comparisons of gene expression between these groups), as the variation between normalization factors generated using two, three, four or five genes was very small. Furthermore, when comparing NPPB levels between failed and non-failed myocardium, removal of the least stable gene, RNPS1, impacted minimally on normalized NPPB levels, suggesting that TPT1 and EEF1A1 may be sufficient for normalization. This reflects the uniformity in expression stability across the top-ranked genes (Figure 2) and is equivalent to using a pairwise variation threshold of 0.23.

Several studies have demonstrated how a single, unvalidated reference gene can generate biased results if it is itself altered by the experimental conditions [5,51-53], In contrast to GAPDH, the expression levels of TPT1 and EEF1A1 did not differ significantly between failed and non-failed human myocardium samples, and they were ranked as being considerably more stably expressed. However, because expression of both genes tended to be lower in failed myocardium compared with non-failed myocardium, TPT1 and EEF1A1 require further validation in a larger sample to confirm that their expression levels do not differ between these groups. Normalizing NPPB expression to GAPDH alone gave a greater difference in NPPB levels between failed and non-failed myocardium than the data normalized to TPT1 and EEF1A1. However, the expression levels of GAPDH were lower in failed heart samples compared with healthy hearts, and this will have artificially skewed the data. Normalizing NPPB expression to TPT1 and EEF1A1 gave a modestly significant difference in NPPB levels between failed and non-failed myocardium that is more likely to reflect the true level of expression of NPPB in these two groups. In particular our finding that GAPDH is differentially expressed in failing and non-failing myocardium has implications for previous RT-qPCR studies of human myocardium that have used GAPDH for normalization without validation.

We have identified reference genes for both failing and non-failing human myocardium that have significantly improved stability compared with the commonly used reference gene, GAPDH (SRP14 and TPT1 for analysis within failed or non-failed tissue and TPT1 and EEF1A1 for comparisons of gene expression between these groups). Our findings suggest that routine normalization of RT-qPCR data in human myocardium with GAPDH should be avoided as it was the least stably expressed of all genes tested. This highlights the importance of validating reference genes for normalization of RT-qPCR data. The reference genes identified in this study will enable more reliable interpretation of RT-qPCR results in these tissues. Whilst TPT1 and EEF1A1 require further validation in a larger study, these findings serve as a basic guideline for reference gene selection in human myocardium.

## Conclusion

This study has identified a strategy for normalization of cardiac gene expression involving two reference genes, SRP14 and TPT1, that in combination may have broad application for accurate and reliable normalization of RT-qPCR data within failed human myocardium and non-failed human myocardium separately. In addition, for comparisons of gene expression between failed and non-failed human myocardium TPT1 and EEF1A1, in combination, may provide a more reliable reference than GAPDH for normalization of RT-qPCR data for these analyses.

## Abbreviations

cDNA: complementary deoxyribonucleic acid; dsDNA: double stranded deoxyribonucleic acid; EEF1A1: eukaryotic elongation factor 1A1; GAPDH: glyceraldehyde-3-phosphate dehydrogenase; GEO: Gene Expression Omnibus; mRNA: messenger ribonucleic acid; NCBI: National Center for Biotechnology Information; NPPB: brain natriuretic peptide precursor; PCR: polymerase chain reaction; RNA: ribonucleic acid; RNPS1: ribonucleic acid binding protein S1; RPL13A: ribosomal protein L13a; RPL22: ribosomal protein L22; RPL23A: ribosomal protein L23a; RPL41: ribosomal protein L41; RPS4X: ribosomal protein S4: X-linked; RT-qPCR: real-time polymerase chain reaction; SRP14: signal recognition particle 14; TCTP: translationally controlled tumor protein; TPT1: tumor protein: translationally-controlled 1.

## Competing interests

The authors declare that they have no competing interests.

## Authors' contributions

APP was involved in the conception and design of the study, performed the gene expression studies and statistical analysis, and wrote the first draft of the manuscript. LJE participated in the data acquisition and provided critical revision of the manuscript. MAB performed the bioinformatics analysis and provided critical revision of the manuscript. CSM was involved in the conception, design and coordination of the study and management of the Cleveland heart tissue bank. WES oversaw tissue acquisition and data coordination. RWT was involved in the conception and coordination of the study and acquisition of funding. AMR was involved with acquisition of funding and provided critical revision of the manuscript. CMF contributed to the study design and performed statistical analysis. VAC was involved in the conception, design and coordination of the study, acquisition of funding, interpretation of the data and provided critical revision of the manuscript. All authors read and approved the final manuscript.

## Pre-publication history

The pre-publication history for this paper can be accessed here:



## Supplementary Material

Additional file 1 **Selection of candidate reference genes.** Expression levels of probe sets for 50 genes previously used or recommended for use as reference genes from publically available Affymetrix gene expression profiles of left ventricle myocardium from 195 heart transplant recipients and 16 unmatched heart donors [11] (GEO accession GSE5406, ). RPL13A, EEF1A1 and RNPS1 (shown in red) were selected as candidate reference genes from this analysis, based on their high abundance and consistent expression across the majority of probes. Affymetrix probe set nomenclature is preceded by the gene symbol. Boxes indicate median and interquartile range, whisker length is 150% of the interquartile range. Observations beyond the whiskers are denoted by open circles. Click here for file
