# Supplementary material for: Genomic selection of reference genes for real-time PCR in human myocardium
Source: BMC Med Genomics. 2008 Dec 29;1:64. doi: 10.1186/1755-8794-1-64 (PMC2632664; doi:10.1186/1755-8794-1-64)
Supplement: Additional file 1 — Selection of candidate reference genes. Expression levels of probe sets for 50 genes previously used or recommended for use as reference genes from publically available Affymetrix gene expression profiles of left ventricle myocardium from 195 heart transplant recipients and 16 unmatched heart donors [11] (GEO accession GSE5406, ). RPL13A, EEF1A1 and RNPS1 (shown in red) were selected as candidate reference genes from this analysis, based on their high abundance and consistent expression across the majority of probes. Affymetrix probe set nomenclature is preceded by the gene symbol. Boxes indicate median and interquartile range, whisker length is 150% of the interquartile range. Observations beyond the whiskers are denoted by open circles. [file 1755-8794-1-64-S1.pdf]

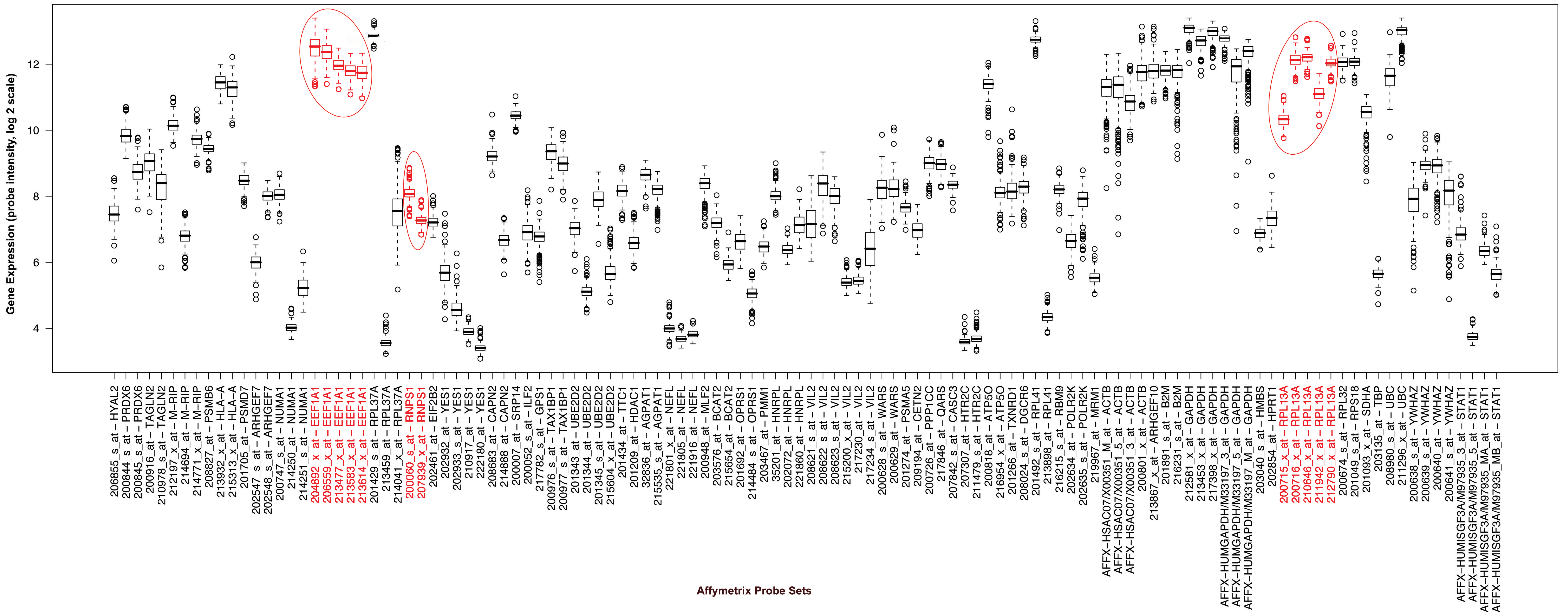

Gene Expression (probe intensity, log 2 scale)
4
6
8
10
12
Affymetrix Probe Sets
206855_s_at – HYAL2
200844_s_at – PRDX6
200845_s_at – PRDX6
200916_at – TAGLN2
210978_s_at – TAGLN2
212197_x_at – M-RIP
214694_at – M-RIP
214771_x_at – M-RIP
208827_at – PSMB6
213932_x_at – HLA-A
215313_x_at – HLA-A
201705_at – PSMD7
202547_s_at – ARHGEF7
202548_s_at – ARHGEF7
200747_s_at – NUMA1
214250_at – NUMA1
214251_s_at – NUMA1
204892_x_at – EEF1A1
206559_x_at – EEF1A1
213477_x_at – EEF1A1
213583_x_at – EEF1A1
213614_x_at – EEF1A1
201429_s_at – RPL37A
213459_at – RPL37A
214041_x_at – RPL37A
200060_s_at – RNPS1
207939_x_at – RNPS1
202461_at – EIF2B2
202932_at – YES1
202933_s_at – YES1
210917_at – YES1
222180_at – YES1
208683_at – CAPN2
214888_at – CAPN2
200007_at – SRP14
200052_s_at – ILF2
217782_s_at – GPS1
200976_s_at – TAX1BP1
200977_s_at – TAX1BP1
201343_at – UBE2D2
201344_at – UBE2D2
201345_s_at – UBE2D2
215604_x_at – UBE2D2
201434_at – TTC1
201209_at – HDAC1
32836_at – AGPAT1
215535_s_at – AGPAT1
221801_x_at – NEFL
221805_at – NEFL
221916_at – NEFL
200948_at – MLF2
203576_at – BCAT2
215654_at – BCAT2
201692_at – OPRS1
214484_s_at – OPRS1
203467_at – PMM1
35201_at – HNRPL
202072_at – HNRPL
221860_at – HNRPL
208621_s_at – VIL2
208622_s_at – VIL2
208623_s_at – VIL2
215200_x_at – VIL2
217230_at – VIL2
217234_s_at – VIL2
200628_s_at – WARS
200629_at – WARS
201274_at – PSMA5
209194_at – CETN2
200726_at – PPP1CC
217846_at – QARS
207842_s_at – CASC3
207307_at – HTR2C
211479_s_at – HTR2C
200818_at – ATP5O
216954_x_at – ATP5O
201266_at – TXNRD1
208024_s_at – DGCR6
201492_s_at – RPL41
213898_at – RPL41
216215_s_at – RBM9
202634_at – POLR2K
202635_s_at – POLR2K
219967_at – MRM1
AFFX–HSAC07/X00351_M_at – ACTB
AFFX–HSAC07/X00351_5_at – ACTB
AFFX–HSAC07/X00351_3_at – ACTB
200801_x_at – ACTB
213867_x_at – ARHGEF10
201891_s_at – B2M
216231_s_at – B2M
212581_x_at – GAPDH
213453_x_at – GAPDH
217398_x_at – GAPDH
AFFX–HUMGAPDH/M33197_3_at – GAPDH
AFFX–HUMGAPDH/M33197_5_at – GAPDH
AFFX–HUMGAPDH/M33197_M_at – GAPDH
203040_s_at – HMBS
202854_at – HPRT1
200715_x_at – RPL13A
200716_x_at – RPL13A
210646_x_at – RPL13A
211942_x_at – RPL13A
212790_x_at – RPL13A
200674_s_at – RPL32
201049_s_at – RPS18
201093_x_at – SDHA
203135_at – TBP
208980_s_at – UBC
211296_x_at – UBC
200638_s_at – YWHAZ
200639_s_at – YWHAZ
200640_at – YWHAZ
200641_s_at – YWHAZ
AFFX–HUMISGF3A/M97935_3_at – STAT1
AFFX–HUMISGF3A/M97935_5_at – STAT1
AFFX–HUMISGF3A/M97935_MA_at – STAT1
AFFX–HUMISGF3A/M97935_MB_at – STAT1
